# Autoimmune encephalitis: recent clinical and biological advances

**DOI:** 10.1007/s00415-023-11685-3

**Published:** 2023-04-28

**Authors:** James A. Varley, Christine Strippel, Adam Handel, Sarosh R. Irani

**Affiliations:** 1 Department of Brain Sciences, Charing Cross Hospital, Imperial College London, Fulham Palace Road, London, W6 8RF UK; 2 Oxford Autoimmune Neurology Group, Nuffield Department of Clinical Neurosciences, University of Oxford, Level 3, West Wing, John Radcliffe Hospital, Oxford, OX3 9DS UK; 3 Department of Neurology, John Radcliffe Hospital, Oxford University Hospitals, Oxford, OX3 9DU UK

**Keywords:** Aautoimmune, Encephalitis, Limbic, Immunotherapy, LGI1, NMDAR

## Abstract

In 2015, we wrote a review in The Journal of Neurology summarizing the field of autoantibody-associated neurological diseases. Now, in 2023, we present an update of the subject which reflects the rapid expansion and refinement of associated clinical phenotypes, further autoantibody discoveries, and a more detailed understanding of immunological and neurobiological pathophysiological pathways which mediate these diseases. Increasing awareness around distinctive aspects of their clinical phenotypes has been a key driver in providing clinicians with a better understanding as to how these diseases are best recognized. In clinical practice, this recognition supports the administration of often effective immunotherapies, making these diseases ‘not to miss’ conditions. In parallel, there is a need to accurately assess patient responses to these drugs, another area of growing interest. Feeding into clinical care are the basic biological underpinnings of the diseases, which offer clear pathways to improved therapies toward enhanced patient outcomes. In this update, we aim to integrate the clinical diagnostic pathway with advances in patient management and biology to provide a cohesive view on how to care for these patients in 2023, and the future.

## Introduction

Since our last review of autoimmune encephalitis due to neuroglial surface targeted (NSAbs) antibodies [1], nascent research into these disorders has taken significant phenotypic, therapeutic, and biological strides. These immunotherapy-responsive conditions are typically associated with autoantibodies which target the extracellular domain of a central nervous system (CNS) cell surface protein. By contrast, most of the, predominantly paraneoplastic, syndromes characterized by ‘onconeuronal’ antibodies (Hu, Yo, Ma, Ri, and CV2/CRMP5) directed against intracellular antigens show a limited response to immunotherapy [2, 3]. Due to their inherent treatability, this review predominantly focuses on the ‘not to miss’ NSAb-mediated conditions. It also provides brief updates on two more recently described conditions associated with antibodies against the intracellular targets, glial fibrillary-associated protein (GFAP) and kelch-like protein 11 (KLH-11), both of which also show evidence of immunotherapy responsiveness.

**Fig. 1:**
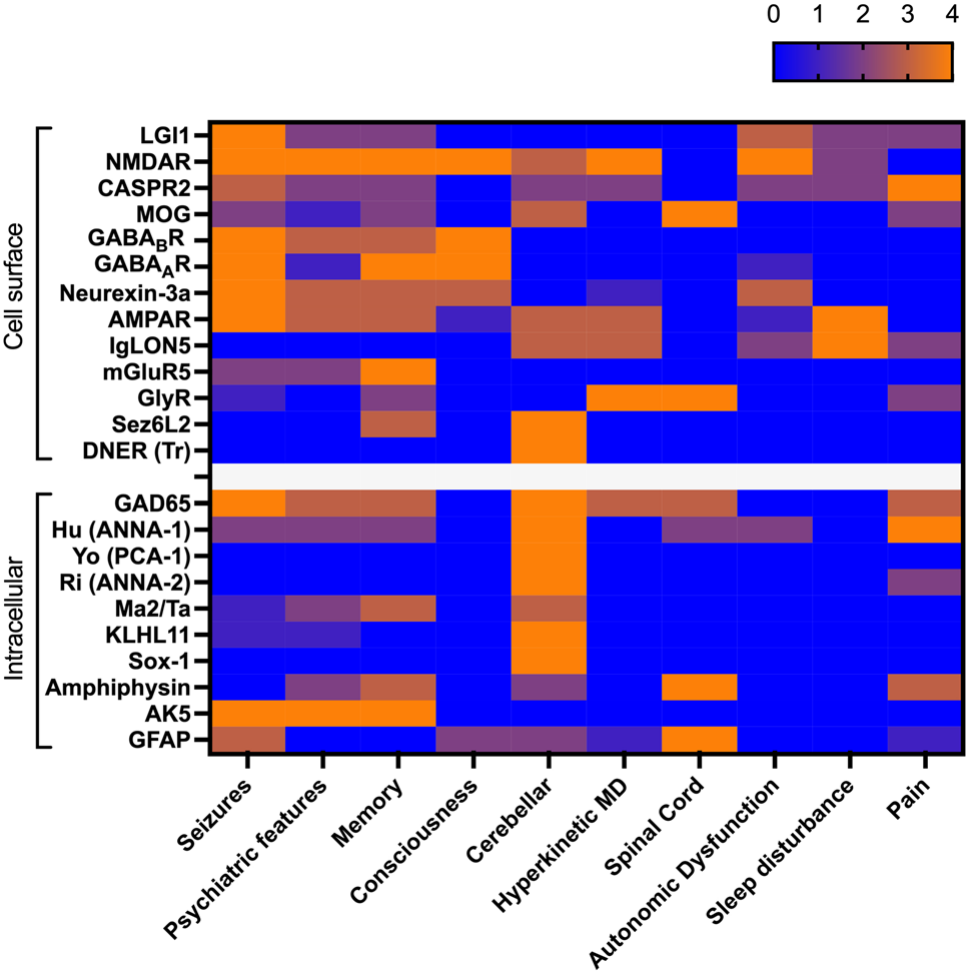
Advances in phenotype. Heatmap illustrating the frequency of autoantibody-associated encephalitis syndromes with frequencies of features from rare or unknown (0 = teal) to common (4 = red). LGI1: leucine-rich glioma-inactivated 1. NMDAR: *N*-methyl-d-aspartate receptor. *CASPR2* contactin-associated protein-like 2, *MOG* myelin oligodendrocyte protein, *GABA*_*B*_*R* γ-aminobutyric acid B receptor, *GABA*_*A*_*R* γ-aminobutyric acid A receptor, *AMPAR* α-amino-3-hydroxy-5-methyl-4-isoxazolepropionic acid receptor, *mGluR5* metabotropic glutamate receptor 5, *GlyR* glycine receptor, *Sez6L2* SEZ6L2, seizure-related 6 homolog like 2, *DNER* delta/Notch-like epidermal growth factor-related receptor, *GAD65* glutamic acid decarboxylase (65 kDa isoform), *ANNA 1/2* anti-nuclear neuronal autoantibody type ½, *PCA* Purkinje cell cytoplasmatic autoantibodies, *KLHL11* kelch-like protein 11, *AK5* adenylate kinase 5, *GFAP* Glial Fibrillary acid protein

In terms of advances, there has been further crystallization of the phenotypes of many of these disorders as well as examples of phenotypic expansion (clinical features of the most common forms are summarized in Fig. 1). Ongoing efforts to improve clinical descriptions aim to facilitate prompt diagnosis and institution of early treatment, which is proven to benefit patients [4, 5]. In parallel, we have learnt more about how patients fare in the longer term, the issues they face in their recovery, and the steps we can take to provide the best possible outcome for them. To this end, there are some innovative immunotherapeutics on the horizon and in clinical trials. In addition, significant progress has been made into understanding the immunological mechanisms underlying autoantibody production in these conditions and how these autoantibodies interact with their antigenic targets to induce neuronal dysfunction. These advances have created potential therapeutic opportunities to intervene directly in disease pathogenesis. Herein, we integrate these clinical and translational observations and explore how they have progressed the field.

### Leucine-rich glioma-inactivated 1 (LGI1)

Patients with LGI1-antibodies represent the commonest form of autoimmune encephalitis, which likely remains under-recognized due to its frequently insidious onset, the subtle focal seizures and its predilection for elderly males, a demographic not traditionally considered to have a primary autoimmune basis for their disease. These patients most commonly present with frequent, focal seizures [6], often the pathognomonic faciobrachial dystonic seizures (FBDS), discussed in more detail in our previous review. Other ictal semiologies have medial temporal lobe predominance and comprise bradycardia, thermal changes [7] or autonomic features such as piloerection [8]. All of these are preferentially sensitive to immunotherapies over anti-seizure medications (ASM).

Crucially, focal seizures precede limbic encephalitis (LE) in around 75% of cases, presenting an opportunity to alter the natural history of the disease [9–11]. The natural history of LGI1-antibody encephalitis appears to be the invariable progression from seizures alone to an established LE [4], with prominent memory disturbance, frequent and ASM-resistant seizures and psychiatric disturbances [12, 13]. As patients progress clinically, their paraclinical investigations become increasingly abnormal, whereas patients with LGI1-antibodies and FBDS alone typically do not have abnormal investigations [4, 12, 13]. Thereafter, increasing cognitive impairment parallels the accumulation of abnormal investigation findings, including hippocampal hyperintensities on T2-weighted MRI, ictal EEG abnormalities, and serum hyponatraemia, due to syndrome of inappropriate anti-diuretic hormone secretion (SIADH). Patients who develop LE are at risk of hippocampal atrophy associated with a fixed memory deficit with concomitant long-term disability [14, 15]. It is this, and a number of other residual cognitive deficits noted in these patients [16], which are potentially avoidable; it appears that early immunotherapy, particularly with corticosteroids [4, 17], may prevent the progression from FBDS to LE. This may be because immunotherapies are the mainstay of treatment for these seizures, and are far more effective than ASMs, or because they have an independent effect on altering disease progression. Overall, these findings emphasize the importance of neurologists being aware of the focal seizures as an early, treatable clinical presentation.

Another striking feature of patients with LGI1-antibodies is that around 25% exhibit adverse reactions toward certain first generation ASMs, specifically carbamazepine and phenytoin, including life-threatening Stevens–Johnson spectrum reactions [4]. This clinical observation led to the suspicion of a potential human leukocyte antigen (HLA) association in this patient group, as discussed below.

### Contactin-associated protein-like 2 (CASPR2)

Patients with CASPR2-antibody-mediated neurological symptoms more often have symptoms affecting the peripheral nervous system, when compared to those with LGI1-antibodies. This was originally appreciated in the form of neuromyotonia (NMT), with peripheral nerve hyperexcitability, which manifests with cramps, stiffness, and fasciculations [18]. NMT can be seen in isolation or in combination with LE, along with a spectrum of prominent autonomic and sleep disturbance in the eponymous Morvan’s syndrome. In Morvan’s syndrome, CASPR2- and LGI1-antibodies often coexist in individual patients [19]. However, most patients with CASPR2-antibodies present with LE but without NMT. This LE can occur with ataxia, other movement disorders and neuropathic pain as additional features [19–22]. Although, as seen very rarely in patients with NMDAR-antibodies, patients with CASPR2-antibodies can infrequently have mono/oligo-symptomatic presentations: for example, isolated epilepsy, ataxia, or cognitive impairment [12, 19, 23].

Recent work has highlighted that movement disorders can be seen in ~ 35% of patients with CASPR2-antibodies, versus 4% with LGI1-antibodies [24]. A variety of movement disorders are appreciated including prominent ataxia, myoclonus, and tremor, as well as some more distinctive subtypes, including episodic ataxia, paroxysmal orthostatic segmental myoclonus of the legs, and continuous segmental spinal myoclonus [24, 25]. These create a significant, sometimes clinically challenging, overlap with functional neurological presentations.

Seizure semiology in patients with CASPR2-antibodies at presentation are predominantly focal (70%), with impaired awareness and limited motor components, or even limited to sensory seizures. Around 50% of patients go on to develop bilateral tonic clonic seizures later in their illness [26].

The pain in patients with CASPR2-antibodies has been recently detailed [27]. Significant, typically neuropathic, pain can be seen in 52% of patients with CASPR2-antibodies, compared to only 19% of patients with LGI1-antibodies. Patients were found to have normal nerve conduction studies but reduced intraepidermal nerve fiber densities. Pain in those with CASPR2-antibodies responded less well to immunotherapy than in LGI1-antibody patients, identifying an important unmet need in the current immunotherapy regimes. In vitro work demonstrated serum CASPR2-antibodies bound unmyelinated human sensory neurons and rat dorsal root ganglia, offering a biological substrate for the pain. In a cohort focusing on acquired neuromyotonia, secondary to either LGI1- and CASPR2- (plus now likely clinically irrelevant double-negative VGKC-) antibodies, pain was reported by patients as a common feature [28].

### *N*-methyl-d-aspartate receptor-antibody encephalitis (NMDAR-AbE)

In NMDAR-AbE, neuropsychiatric symptoms are often preceded by viral prodromal symptoms, such as headache, fever, myalgia, and coryza, which usual occur around 4-14 days  prior to disease onset. This is followed by an acute neuropsychiatric presentation with subsequent seizures and cognitive impairment. Around 1–4 weeks later, patients commonly develop a characteristic movement disorder [29], dysautonomia, and coma [30], which often precipitate intensive-care unit admission.

These features of NMDAR-AbE have been endophenotyped in further detail, highlighting some distinctive characteristics. Studies of the associated movement disorder show that it is a common feature, can be the presenting sign in children and adolescents [5], and, along with the neuropsychiatric symptoms, can be prolonged, continuing for a median of 112.5 days in this cohort [31]. Descriptions of the NMDAR-AbE-associated movement disorder by expert raters required a wide range of descriptors to capture its full phenomenology, characterized as an unusual combination of stereotypies, chorea, and dystonia not classically observed together in the other movement disorders [31, 32].

The prominent psychiatric symptoms associated with NMDAR-AbE are often the initial features of this illness in adults [30], with as many as 77% of patients first presenting to psychiatrists in some early series [33]. A systematic review encompassing 464 patients with NMDAR-AbE demonstrated the complexity of NMDAR-AbE-associated psychopathology, with the otherwise unusual combination of behavioral (68%), psychotic (67%), and mood (47%) features coexisting in individual patients. This constellation was not effectively captured by individual DSM-V or ICD-10 criteria for primary psychiatric syndromes [34]. In the future, we anticipate these studies, and similar studies expanding on this work, will aid clinicians in even earlier identification and treatment of this condition. This is likely to assist clinicians in starting treatment even prior to the availability of autoantibody results, something which is becoming our common practice and is associated with improved patient outcomes [5].

A long-standing clinical question concerns whether NMDAR-AbE can present as isolated psychiatric syndrome. This hypothesis seems attractive, since glutamatergic dysfunction is considered key to schizophrenia pathogenesis and disruption of NMDAR signaling via a variety of modalities capable of producing psychotic symptoms [35]. Furthermore, serum NMDAR-Abs were detected in patients with early, or first episode, presentations of psychosis [35]. However, some conflicting results have been published, with some investigators finding (typically small) differences between the prevalence of serum NMDAR-antibodies in patients with first-episode psychosis and controls [36–38], and others finding no differences [39, 40]. These studies, in turn, have propagated several more comparisons, which exhibit important differences in assay methodologies, timing of sample collection, and materials tested. While methodological differences may largely account for the heterogeneity in study findings, overall, it appears that CSF NMDAR-antibodies, ideally tested using a combination of tissue immunochemistry and live cell-based assays, provide very specific (albeit not 100%) diagnostic information. Indeed, positivity for this combination has not been found in large series of patients with schizophrenia [41] or first-episode psychosis [42, 43]. Overall, these studies suggest that while mono- and oligo-symptomatic NMDAR-AbE presentations (such as early forms of psychosis) may occur, typically in relapsing NMDAR-AbE, they represent the vast minority of cases. Rather, in most presentations, an abrupt onset together with the canonical diffuse and multimodal clinical features described above should be considered as the core features of NMDAR-AbE.

### γ-Aminobutyric acid A receptor (GABA_A_-R)

Patients with GABA_A_-R antibodies are more recently described. Early series described patients presenting with prominent seizures, which can be a potential cause of treatment resistant status epilepticus requiring intensive care. Patients were also frequently found to have memory impairment, disorientation, and psychiatric features [44, 45]. More recent series emphasize that, compared to other neuronal-surface antibody (NSAb)-associated encephalitides, the MRI imaging is abnormal in around 80% of cases [46] and is, in our experience, a highly specific diagnostic feature. Most patients have widely distributed, cortical–subcortical T2/FLAIR hyperintensities in predominantly the temporal or frontal lobes with limited diffusion restriction and are dynamic, responding to immunotherapy and relapses [47]. These imaging findings can be a clue to guide early autoantibody testing and administration of immunotherapies in the correct clinical context.

### IgLON5

Much interest was generated from the description of patients with antibodies to IgLON5 [48], principally because this was the first NSAb-associated disease with a prominent and intriguing overlap between a neurodegenerative and immune-mediated disease. The clinical tempo and phenotype of patients with IgLON5-antibodies favor neurodegeneration, confirmed with prominent tau deposition on neuropathology. However, the autoantibodies target the extracellular domain of a neuronal protein, suggesting direct causality. The clinical presentation includes early prominent sleep disorders in REM and non-REM stages with dream re-enactment, stridor, a complex set of movement disorders, dysautonomia, and bulbar involvement. An important mimic, although reasonably consistently differentiated given the nature of the sleep disorder, is progressive supranuclear palsy [49]. Similar to the NMDAR-AbE movement disorder, that of IgLON5-antibodies spans a wide variety of clinical phenomenologies, including gait instability, chorea, bradykinesia, dystonia, tremor, myoclonus, hyperekplexia, and cramps/fasciculations [50, 51].

In these patients, a combination of bulbar, respiratory, and autonomic involvement may explain why many of the initially reported cases died suddenly and unexpectedly, and progressed to autopsy. These patients were initially felt not to respond to immunotherapy [48], consistent with a dominant neurodegenerative disorder. However, more optimistically, later series, the largest of which contained 53 patients, have demonstrated that 75% of treated patients show some response to immunotherapies, including first- and second-line agents [50, 52]. Our clinical experience has been of temporary unequivocal partial improvement or stabilization with the first-line immunotherapies, which is then followed by occasional stabilization but, more commonly, subsequent deteriorations, which can be very challenging to manage. Hence, in this condition, it remains to be observed whether suppression of the probable immunological effector limb is sufficient to halt, or even reverse, the longer-term disease process.

### Myelin oligodendrocyte protein (MOG)

MOG antibody-associated disorder (MOGAD) was originally identified as a CNS demyelinating disorder in patients with neuromyelitis optica (NMO) without aquaporin-4 (AQP4) antibodies [53, 54]. Typical patients were reported to have either optic neuritis or myelitis or a combination, including a near-synchronous onset of both. More recently, around 50% of cases with acute disseminated encephalomyelitis (ADEM) have been shown to have MOG-antibodies, and these antibodies are also detected in patients who have cortical encephalitis with leptomeningeal inflammation [55–58]. Patients with cortical encephalitis secondary to MOGAD most often present with seizures, which often have a focal unilateral limb onset before becoming generalized, as well as headache, fever, and occasional signs of cerebral irritation, such as confusion, lethargy, and memory impairment [56, 57, 59].

In MOGAD cortical encephalitis, MRI reveal focal cortical FLAIR hyperintensities with some associated sulcal FLAIR hyperintensity and meningeal enhancement. CSF pleocytosis is seen in most patients with this syndrome, often without oligoclonal bands or other biochemical signs of inflammation [56, 57, 59]. The histopathology of MOGAD cortical encephalitis demonstrates subpial lesions with perivenous demyelination, which are also seen in cases of acute disseminated encephalomyelitiss [59, 60], as well as microglial reactivity and inflammatory infiltrates in the meninges or around blood vessels [59]. The syndrome often responds highly effectively to corticosteroids with or without other first-line immunotherapies. Yet, relapses are seen in up to 40% of cases, leaving unanswered questions about whether repeat corticosteroid courses, steroid-sparing agents, or rituximab should be considered more routinely in management of the first episode.

### GFAP

GFAP-antibodies have recently been described in a group of immunotherapy-responsive syndromes, often with meningoencephalomyelitis as the classical, overarching manifestation [61]. In the largest series to date (*n* = 102) [62], a viral prodrome was seen in the majority, 94% had either meningitis, encephalitis or myelitis, around 30% had concurrent AQP4- or NMDAR-antibodies, and around 30% had an underlying neoplasia. GFAP-antibody testing in the CSF is key to establishing the disease diagnosis and, as GFAP is an intracellular protein, these antibodies are likely biomarkers of an underlying process rather than being directly pathogenic [63].

As a diagnostic aide, MRI imaging is almost always abnormal in this condition [62, 64]. Characteristic brain contrast enhancement identified a linear, radial perivascular pattern through the periventricular white matter, though there can also be leptomeningeal enhancement. In the spinal cord, there is often longitudinally extensive myelitis with central enhancement. In addition, contrast enhancement is observed in around two-thirds of cases. CSF constituents are abnormal in the majority, with a lymphocyte pleocytosis (mean cell count 80 /ul), raised protein, and oligoclonal bands present in ~ 50% of cases. EEG can be abnormal with generalized slowing. Patients respond variably to treatment, but 73% with a meningoencephalomyelitis were shown to respond to the first-line immunotherapies, while the second-line agents were needed in refractory or relapsing cases, which should also prompt a more detailed search for neoplasms [62–64]. One key question is whether the frequent coexistence of GFAP-antibodies with antibodies of other reactivities suggests that GFAP-antibodies are an epiphenomenon generated as part of epitope spread or are an indication of pathogenic GFAP-directed T-cell immunity.

### KLH-11

Antibodies to KLHL-11 were described in 2019 in a small series of patients with testicular seminoma and a rhomboencephalitis lacking the Ma-2 antibodies classically expected in this context [65]. Interestingly, the neurological syndrome preceded the seminoma diagnosis in 9 of 13 patients (69%). A larger retrospective series showed this antibody only in men, presenting with a rhomboencephalitis: 83% were associated with evidence of a testicular germ cell tumors (including radiological spontaneous regression) and there was suggestion of an over-representation of HLA-DQB1*02:01 and HLA-DRB1*03:01 alleles [66]. A 58% response to immunotherapy ± cancer treatment indicated that autoantibodies to this intracellular antigen may show treatment responses.

In contrast, a different cohort study reported an equal sex distribution but a similar association with tumors (72%), which were predominantly benign teratomas of the testis but also seminomas or mixed germ cell tumors. Clinically, 41% had an ataxic or brainstem predominant syndrome and this subgroup was enriched for a tumor association (85%) [67].

In summary, this novel antibody should be tested in patients, especially men, presenting with brainstem symptoms or ataxia and, if positive, should trigger a high suspicion for a tumor as well as a trial of immunotherapy. Further work is required to understand fully the immunobiology of KLHL-11 encephalitis.

## Advances in the underlying immunobiology

**Fig. 2:**
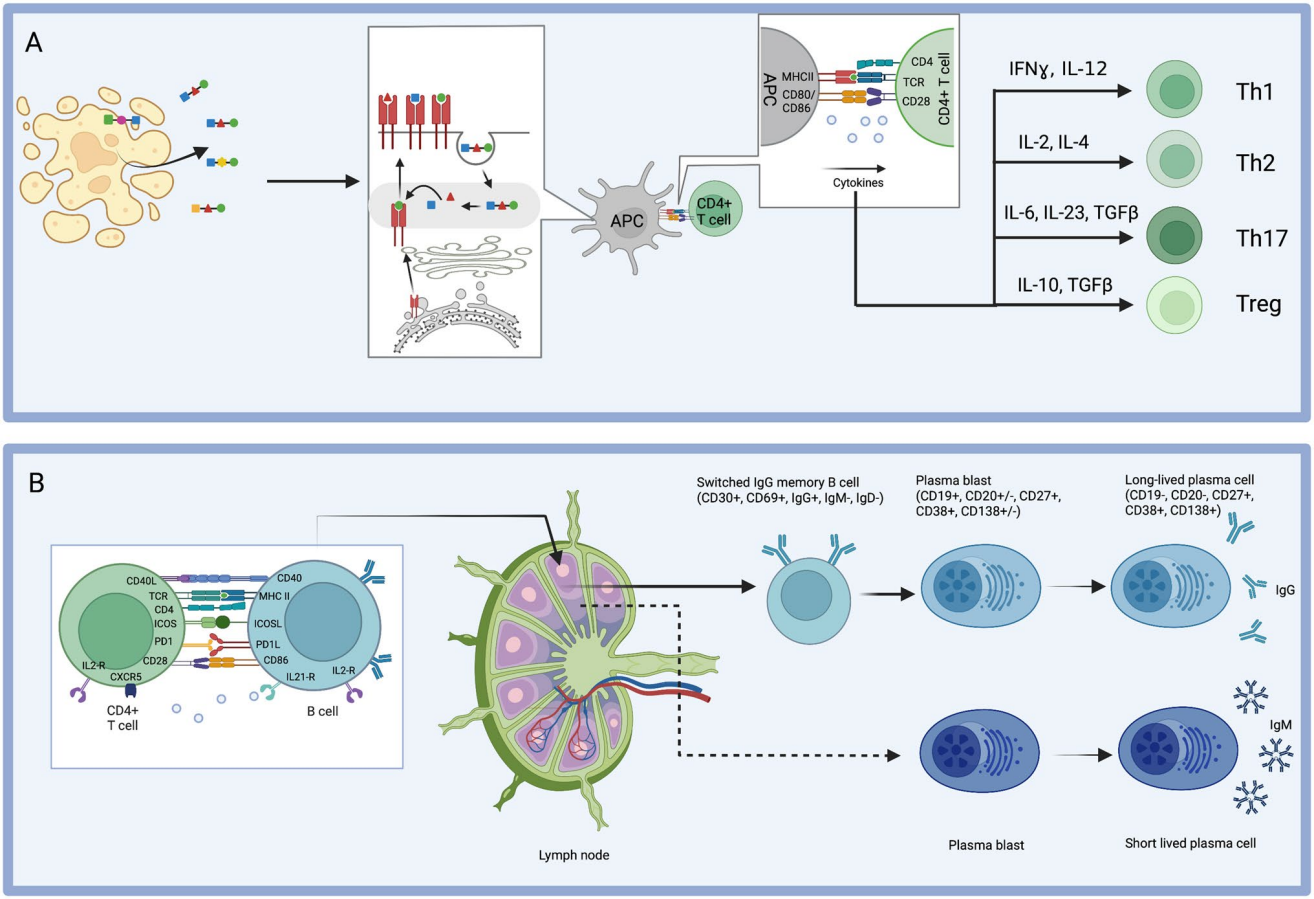
Immunological Aspects. **A** The generation of antigen-specific T cells involves antigen presentation by professional antigen-presenting cells (APC) including dendritic cells, macrophages, and B cells. The APC take up antigens and process them into MHC II-antigen complexes, which are displayed on the cell surface. At the “immunological synapse” between APC and T cell, classically three signals are exchanged: The first signal is the T-cell receptor (TCR)–MHCII complex interaction; the second signal is transmitted via other surface proteins (e.g., CD80/86 and CD28) and the third include soluble factors (e.g., various cytokines), which help polarize T-cell subtypes. Adapted from Roche et al. [69] and Sharabi et al. [70]. **B** T and B cells interact tightly to generate soluble autoantibodies. Cognate T-cell encounters enable B cells to mature into IgM producing, short-lived plasma cells (SLPC), often bypassing germinal centres (GC). The GC response results in higher affinity antibodies and immunological memory. Activated B cells undergo clonal expansion and somatic hypermutation, and the latter matures and improves the strength of the B cell for its antigen. The resulting memory B cells and plasmablasts exit the GC and home to their survival niches as long-lived plasma cells (LLPC). Adapted from Sun et al. [68] and Zografou et al. [71] and Stebegg et al. [72]

Since their original clinical descriptions, our understanding of the immunobiology of the diseases summarized in the previous section has advanced considerably [68]. Selected highlights are provided below and summarized in Fig. 2.

### In the periphery

Two key goals in all autoantibody-mediated diseases are to accurately describe the immunological compartments where self-targeted B-cell receptors (BCRs; the B-cell surface bound antibody) are generated and escape mechanisms of immune tolerence, and to understand mechanisms by which the autoantibody response is propagated. Both show clear relevance to the selection of therapeutics and are often modeled by two broad immunological schemata [73, 74]. The first suggests that, throughout disease, ongoing germinal center reactions generate autoantigen-reactive B and antibody-secreting cells. The other, somewhat opposing, model is of a remote breakdown in tolerance which, via a brief germinal center reaction, produces long-lived plasma cells (LLPC), which likely reside in bone marrow or CNS niches. This second model is thought to be how lifelong immunity is acquired from childhood vaccinations and, from a therapeutic perspective, is important as LLPCs are non-proliferative and do not express CD20. Therefore, these cells are theoretically insensitive to drugs, such as azathioprine, mycophenolate mofetil, or rituximab. By studying peripheral blood mononuclear cells (PBMCs) from patients with NMDAR-AbE, Makuch et al. [75] were able to demonstrate that patient circulating B cells frequently generate NMDAR-reactive antibodies, at levels proportional to the corresponding patient’s serum NMDAR-antibody titres. This suggested an ongoing immunological process, and argued against predominantly LLPC-driven mechanisms. Similarly, NMDAR-reactive IgMs were found in the patient sera at many timepoints during the course of the disease, again supporting evidence of ongoing, active immune reactions. In addition, the key tumor association in this condition, an ovarian teratoma, pointed more directly toward the presence of active germinal centers. Teratoma tissue contained dense clusters of B and T cells, with the NR1 (‘immunodominant’) autoantigen in close apposition to follicular dendritic cells, lymphatic structures, and high endothelial vasculature [76]. These observations were consistent with the presence of an intratumoral tertiary lymphoid structure, with germinal center-like capacity. Furthermore, in culture, it has been shown that these lymphocytes had the capacity to produce NMDAR-specific antibodies. This provided evidence of an active, NMDAR-focused, tumor-based germinal center reaction in NMDAR-AbE.

However, only around 30% of patients with NMDAR-AbE have ovarian teratomas. Hence, the search for germinal centers was extended with a pioneering approach to directly sample lymphocytes from patient cervical lymph nodes [76], which represent the likely anatomical site of CNS lymphatic drainage [77]. Culture of these lymphocytes was found to produce NMDAR-IgG, particularly in patients with higher serum NMDAR-antibody levels and the highest levels of the germinal center marker CXCL13 [76]. Taken together, both patient cervical lymph nodes and ovarian teratomas provide evidence of active NMDAR-antibody generating germinal center reactions.

In a related disease, neuromyelitis optica spectrum disorders (NMOSD) associated with aquaporin-4 antibodies, there is evidence to suggest that patients may have an intrinsic predisposition to the entry of autoantigen-reactive B cells into germinal centers. Naïve B cells (which are not thought to enter germinal centers) from patients but not healthy controls were found to carry AQP4-reactivities. This finding suggests that naïve B cells, newly emerging from the bone marrow, may contribute a proportion of the ongoing immune response in patients [78]. It remains to be ascertained whether this can be confirmed in future studies and whether this principle translates to autoimmune encephalitis.

Germinal centers are noted for their ability to imprint BCRs with somatic hypermutations, which classically both increase antibody affinities and generate diverse epitopes. Indeed, peripheral autoantigen-reactive B cells in patients with LGI1-antibody encephalitis identified heterogenous and abundant patterns of hypermutation, targeting of multiple epitopes within the two major domains of LGI1, and found that some LGI1-reactive monoclonal antibodies with high affinity BCRs were able to cause pathology when injected into mice [79]. Again, and taken together with the fundamental observation that these LGI1-reactive memory B cells were detected in the periphery, these findings all support a role for germinal centers in producing these autoreactive B cells.

The evaluation of a biological role for germinal centers is made even more clinically relevant by a recent therapeutic observation. While several studies had suggested that lymph nodes are often resistant to rituximab, an immunotherapeutic agent directed against CD20 [80, 81], a study of fine-needle aspirations in humans identified marked sensitivity of cervical lymph -node B cells to rituximab, with a lymph-node specific reduction in autoantibody levels [82].

Germinal centers involve direct interactions of B and T cells and, in diseases whose core feature is autoantibody production, CD4 T cells are particularly implicated as key helpers of—cell activation, division, and differentiation. Class II HLA molecules are involved in presenting peptide antigens to CD4 + helper T cells. Hence, it appears biologically intuitive that strong class II HLA association have been identified in some autoantibody-mediated diseases, especially in patients with antibodies to LGI1, CASPR2, and IgLON5 [83–85]. The HLA-DRB1*07:01 gene is carried by ~ 95% of patients with LGI1-antibodies, and homozygosity is associated with a doubling of risk, a dose–response suggesting causality [86]. Around 50% of patients with CASPR2-antibodies carry a specific HLA allele, DRB1*11:01, which increases to ~ 90% in patients with CASPR2-antibodies in the context of limbic encephalitis. In addition, ~ 90% of patients with IgLON5-antibodies carry both HLA-DRB1*10:01 and HLA-DQB1*05:01 alleles. These collective genetic findings indicate that individual antigens bind to specific HLA alleles which are a crucial step in the development of their respective autoantibody-mediated conditions. These HLA associations are also in keeping with in vitro data, which suggested that conditions mimicking T-cell help were most likely to lead to specific antibody production [75, 78].

However, most autoantibody-mediated diseases lack strong HLA associations and so these genetic links do not appear necessary for generation of all autoantibodies, suggesting that alternative immunological mechanisms await discovery.

### In the CNS

If and how the peripheral immune response migrates to the CNS is another intriguing, and therapeutically relevant, question. The log-fold higher absolute levels of autoantibodies in serum versus the CSF might suggest that the autoantibody generation begins in the periphery. However, when normalized for total IgG levels in each compartment, intrathecal synthesis is often apparent: this is a biochemical measure which suggests the presence of autoantigen-reactive B cells in the CSF.

One study, elegantly proving this concept, cloned and expressed individual BCRs from the CSF memory and antibody-secreting B cells in patients with NMDAR-AbE [87]. Around 10% of BCRs were found to react to NMDARs and their mutational patterns showed both immunoglobulin class switching and somatic hypermutation, suggesting that these B cells had undergone affinity maturation within a germinal center. However, intriguingly, a minority were non-mutated and essentially germline encoded, perhaps indicating a more fundamental genetic predisposition to this rare illness. The same group studied CSF B cells in patients with LGI1- and GABA_A_R-antibody encephalitis [88], and found a high frequency of highly mutated autoantigen-reactive BCRs in both conditions [89]. While these collective studies have identified abundant intrathecal autoantigen-reactive B cells, the directionality of these cells is unknown. Yet, BCRs expanded in CSF clones do appear to be found within the BCR repertoire of peripheral B cells, suggesting a dynamic exchange between B cells in these two compartments [90]. Cross-sectional study designs in further subjects, especially when untreated, could aim to address these questions in the future.

## Advances in the underlying neurobiology

**Fig. 3:**
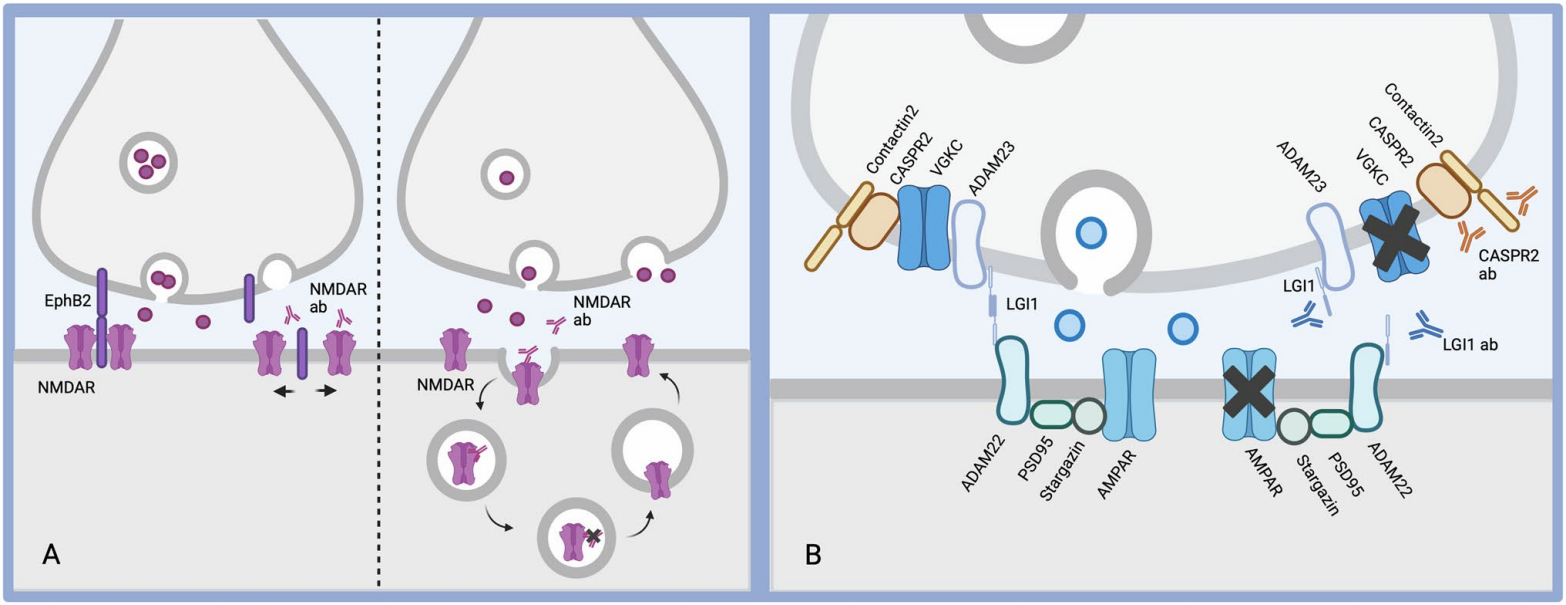
Advances in neurobiology. **A** In NMDAR-antibody encephalitis. Under physiological conditions, NMDARs are organized in post-synaptic clusters and stabilized by ephrin B2 (EphB2). Autoantibodies in NMDAR-antibody encephalitis disrupt the interaction between NMDARs and EphB2, causing a lateral dispersion of the receptors (left panel). They also cross-link NMDARs causing their internalization through endocytosis (right panel). Adapted from Ladépêche et al. [91]. **B** In LGI1- and CASPR2-antibody diseases. Patient symptoms are likely caused by modulation of AMPARs and VGKCs. LGI1-antibodies disrupt binding of LGI1 to ADAM22/23, which reduces post-synaptic AMPA receptors, thus resulting in hyperexcitability. In CASPR2-antibody encephalitis, patient antibodies potentially disrupt the VGKC–contactin–CASPR2 complex, leading to altered excitability. Adapted from Van Sonderen et al. [92]

Alongside advances in our understanding around the immunological mechanisms which give rise to these pathogenic antibodies, advances have also been made in how these antibodies mediate their effects to cause such clinically characteristic phenotypes (Fig. 3).

In NMDAR-AbE, antibodies are reported to bind the extracellular amino-terminal domain of the NR1 subunit. Patient serum and CSF have been shown in vitro to reduce the size of NMDAR clusters giving rise to impaired glutamatergic signaling. This effect is reversible and resolves on removal of the NMDAR-antibodies [87, 93]. The reduction of NMDAR cluster density is likely driven by two phenomena: internalization of NMDARs and their lateral dispersion from key synaptic signaling areas. Internalisation occurs secondary to antibody-mediated endocytosis, due to cross linking of NMDARs. Lateral dispersion may be driven by disruption of the interaction between NMDARs and Ephrin-B2, a receptor tyrosine kinase which binds and phosphorylates NMDARs [94, 95]. Infusion of Ephrin-B2 into animal models of NMDAR-AbE antagonises this effect, with rescue of the clinical phenotype and preservation of NMDAR cluster density [96].

In LGI1- and CASPR2-antibody encephalitis, there is an evolving neuroscientific understanding of the role of AMPA receptors in disease manifestations. Murine mutation of LGI1 impairs AMPAR signaling, likely via disruption of the presynaptic Kv1.1 potassium channels ADAM23 and post-synaptic ADAM22-AMPA receptor complexes [97]. In vitro and in vivo data demonstrated that LGI1-antibodies disrupt the binding of LGI1 to ADAM22/23, leading to a reduction of post-synaptic AMPA receptors with consequent excess network excitability and seizures [98]. More recent work has shown patient-derived LGI1-directed monoclonal antibodies target different domains of LGI1 and can disrupt binding of LGI1 to ADAM22/23 differentially, with resultant impaired long-term potentiation in animal models [79]. In CASPR2-antibody encephalitis, evidence from animal models suggests that similar mechanisms are in operation, with silencing of CASPR2 either genetically or with autoantibodies resulting in altered AMPA receptor function and subsequent disruption of cortical excitatory transmission [99].

## Advances in management

The management of patients with autoimmune encephalitis is an area informed by expert observations, retrospective observational studies and only one randomized controlled trial. This paucity of classically perceived ‘high quality’ evidence is driven mostly by the relatively scarcity of these conditions and the appropriate onus on early treatment, meaning that studies of interventions are necessarily contaminated by administration of prior immunotherapies. Nevertheless, studies examining treatment interventions and patient outcomes have made considerable progress over the last few years.

### Patient outcomes

Understanding longer term issues which patients encounter continues to be an area of active research with important everyday implications. For example, in NMDAR-AbE, it is widely accepted that treatments are generally highly effective. However, detailed neuropsychological assessments at long-term follow-up have demonstrated that ~ 80% of patients had moderate-to-severe cognitive impairment, with prominent deficits in executive function and memory. This challenges the current perception of this disease as showing a complete response to immunotherapy. Furthermore, poor cognitive outcomes were correlated with late treatments, more severe disease and longer duration of acute illness; making the case again for earlier recognition and aggressive treatment [100]. In pediatric NMDAR-AbE [101], attention and fatigue represent key persistent deficits which correlate with quality of life, suggesting that a younger and more neuroplastic brain is insufficient to protect against this disease effect. Similar observations were made in patients with LGI1-antibody encephalitis [102]. Despite good outcomes based on the widely employed modified Rankin Scale, around 80% of patients showed deficits in cognition, mood or fatigue, with only 15% able to return to work. Even the more disease-specific score, Clinical Assessment Scale in Autoimmune Encephalitis (CASE), only revealed a limited quantitative improvement in the patients, perhaps as this scale may be especially valuable in patients with NMDAR-antibody encephalitis over other forms of autoimmune encephalitis [103]. Taken together, these studies suggest that we should collectively consider these to be partially immunotherapy-responsive conditions in which our management currently remains far from ideal. In future studies, more needs to be done to optimize these long-term outcomes for this patient group.

### Monitoring disease

Perhaps surprisingly for diseases conceptualized to have autoantibodies as the main pathological effectors, autoantibody levels are only imperfectly correlated with disease severity, both between and within patients [104, 105]. Therefore, the question arises: how do we accurately monitor patients? Improved monitoring would yield two clear benefits. First, the ability to predict a recrudescence in disease activity, which could be pre-emptively treated. Second, to help provide a rationale for escalating immunotherapies in view of the ongoing disability demonstrated in the above studies. There are several emerging candidates.

Some of these candidates are immunological in nature. For example, in NMOSD, there was a switch of IgG subclasses and increased production of AQP4-specific IgMs around the time of relapses, implying that these features are consistent with new germinal center activity associated with relapses [82]. Also, CXCL13, a chemoattractant reported to mediate recruitment to and retention of B cells within the CSF, has been associated with a limited response to therapy, more clinical relapses and as a biological correlate of intrathecal NMDAR-antibody synthesis [106].

Others reflect damage to the cells targeted by the autoantibodies. In NMOSD, glial fibrillary acidic protein (GFAP) is an emerging biomarker. GFAP is a cytoskeletal protein specifically expressed in astrocytes, which are the cellular targets of pathogenic AQP4 antibodies. Serum GFAP levels are elevated in NMOSD versus healthy controls and other demyelinating diseases and, within patients, elevated GFAP correlates with relapses, relapse severity, and disease severity, and may predict the propensity to relapse at disease onset [107–109]. Furthermore, there is emerging evidence that neurofilament-light chain measurement in CSF is modestly elevated at presentation in LGI1-, CASPR2-, and NMDAR-AbE, but its prospective predictive value remains unproven [110, 111].

Finally, a recent study in five patients highlighted that direct assessments of the target autoantigen may represent a promising approach. Using a PET ligand which targets activated NMDARs, it was observed that patients recovering from NMDAR-AbE showed a reduction in NMDAR density which correlated with time from disease onset. One patient, who was both furthest into their recovery and had no detectable NMDAR-antibodies, had NMDAR densities equivalent to controls [112]. Hence, imaging tools might be useful to monitor in vivo recovery and could inform longer term treatment decisions.

These are all potentially exciting tools. However, currently, none offer well-validated prospective clinical predictive values, and we continue to rely on clinical judgment to direct patient follow-up.

### Immunotherapy

#### Corticosteroids

Cortisol is the endogenous corticosteroid, produced from cholesterol in the adrenal gland, and acts predominantly on glucocorticoid receptors. Synthetic corticosteroids, including prednisolone, methylprednisolone, and dexamethasone, are selected based on a high glucocorticoid preference. The exact mechanisms by which corticosteroids treat autoimmune CNS conditions is unclear but likely to rely on a combination of their pleotropic abilities to decrease blood–brain barrier permeability [113], rapidly suppress (within minutes) transcription of genes encoding pro-inflammatory cytokines, chemokines and cell adhesion molecules [114], repression of key immunomodulatory transcription factors (e.g., NF-κB and AP-1) [115], suppression of myeloid cell function, and induction of apoptosis in lymphocytes [116]. Due to their clear benefits on cognition and seizure frequency in observational studies, corticosteroids are the most frequently employed the first-line treatment for patients [4, 17]. Corticosteroid dosing and weaning regimes vary hugely between physicians treating these conditions and, in our experience, depends on the autoantibody underlying the disorder. For example, in NMDAR-AbE, we have been successful in corticosteroid induction without tapering, but in LGI1-AbE, we have observed that this approach precipitates relapses, often necessitating prolonged courses of corticosteroids in this condition. Unfortunately, corticosteroid side effects are myriad and include insomnia, diabetes mellitus, hypertension, osteopenia, avascular necrosis, muscle atrophy, and psychiatric disorders. Many of these side effects are related to duration of treatment and dose, and particularly affect the more elderly patients, the main group affected by LGI1-antibody encephalitis.

The choice of corticosteroid is also not closely scrutinized in studies, with prednisolone and methylprednisolone empirically reached for on a neurological ward. However, evidence exists that dexamethasone remains at a higher concentration in the CNS for a longer duration [117], as well as having higher glucocorticoid efficacy and less mineralocorticoid activity. Hence, an ongoing challenge is to establish optimum steroid treatment regimens and mitigate many of these common adverse effects.

#### PLEX, IVIG, and Immunoadsorption

PLEX and IVIG are well-established treatments in autoimmune encephalitis and are often used as first-line immunotherapies [4, 118]. There is some evidence that the addition of PLEX to corticosteroids and IVIG offers superior efficacy [119]. Often, there are logistical factors which inform the use of either IVIG or PLEX, including the scarcity of human immunoglobulins, unwillingness from some patients to accept human blood products, access to PLEX, patient ability to tolerate PLEX, including vascular access issues. Nevertheless, IVIG is the only treatment for autoimmune encephalitis with proven efficacy in a randomized controlled trial [120]. Yet, the effect size was small in absolute terms and only just reached significance by comparison to placebo. In our clinical practice, IVIG is very rarely used, as PLEX appears both to generate striking and rapid improvements in many patients and can be performed through peripheral cannulation, making it both efficacious and de-risking it significantly.

As an alternative to PLEX, immunoadsorption was able to clear autoantibodies from both the serum (97%) and CSF (64%) within 4 days and sustained reductions in autoantibody levels seen at 4 weeks, and was associated with improvement in 86% of patients with NSAbs [121]. Furthermore, immunoadsorption offers the opportunity to spare patients exposure to transfusion products when compared with plasma exchange.

#### Steroid sparing agents

The use of agents, such as methotrexate, azathioprine, and mycophenolate mofetil, is influenced by a variety of factors. For example, patients with NMDAR-AbE are often treated with pulsed corticosteroids and PLEX, with a low threshold to escalate to second-line agents, such as rituximab or cyclophosphamide. First- plus second-line therapies can induce prolonged remission in as many as ~ 95% of cases, leaving little role for the use of steroid-sparing agents. Reported relapse rates are higher in patients with LGI1- and CASPR2-antibody-mediated diseases, particularly if corticosteroids are tapered too quickly. Hence, steroid-sparing agents are employed more often in this context. However, in our experience, relapses remain most strongly related to a shorter taper of steroids despite the use of steroid-sparing agents [10].

#### Monoclonal antibodies

As mentioned previously, the chimeric monoclonal antibody directed against the B-cell marker CD20, rituximab, is widely used in autoimmune encephalitis, most commonly at the earliest time points in NMDAR-AbE [122], but also later in the disease course of patients with autoantibodies against LGI1, CASPR2, and GAD. In the largest retrospective cohort study to date, patients with CASPR2-antibody diseases and NMDAR-AbE showed improvements with rituximab, whereas patients with LGI1-antibodies improved similarly with administration of other immunotherapies [122]. Relapse rate was 13% in controls and 5% in cases following rituximab treatment in a pooled analysis of 228 patients with antibodies to NMDAR, CASPR2 and LGI1. Improved treatment efficacy was observed with its early administration [122]. The overall perceived efficacy of rituximab may suggest a limited role for LLPCs in disease propagation. A clinical trial of Ocrelizumab, a humanized anti-CD20, in autoimmune encephalitis failed to meet target enrollment and was discontinued. Recently, inebilizumab, a CD19-targeted monoclonal antibody, has received FDA approval in NMOSD based on a randomized controlled trial of 230 participants [123]. An infection signature in patients with prior rituximab use was noted following treatment with inebilizumab: 18% serious infection vs 10% with no prior rituximab use (though not statistically significant), and so, further evaluation in this setting is ongoing [124]. This drug is being trialed in NMDAR-AbE based on the biological rationale that CD19 is expressed on more B-lineage cells than CD20, both earlier and later in developmental stages.

#### Novel immunotherapies

Given the significant side effect profile of steroids, the lack of a clear role for steroid-sparing agents and our growing knowledge of the underlying immunology of these conditions, it is fortunate that new and more targeted treatments are on the horizon. IL-6 receptor monoclonal antibodies have been used in autoimmune encephalitis refractory to rituximab with encouraging observational results [125]. A humanized IL-6 monoclonal, already approved for use in NMOSD, is currently being assessed in a clinical trial of patients with autoimmune encephalitis secondary to NMDAR- and LGI1-antibodies (https://clinicaltrials.gov/ct2/show/NCT05503264). Bortezomib is a proteasome inhibitor. As the proteosome is most active in antibody-secreting plasma cells, which express very little CD20, it is being trialed in refractory cases of autoimmune encephalitis, where both first- and second-line treatments have failed (https://clinicaltrials.gov/ct2/show/NCT03993262). Finally, monoclonal antibodies directed against the neonatal Fc receptor, FcRN, are being trialed in these conditions. The FcRN constitutively recycle IgG to preserve its half-life. Hence, their blockade markedly reduces antibody levels. These drugs have shown benefits in myasthenia gravis and are currently being trialed in LGI1-antibody encephalitis (https://clinicaltrials.gov/ct2/show/NCT04875975).

## Conclusions

Since 2015, the field of autoimmune encephalitis has witnessed a number of advances in our understanding of the underlying biology and the correlation of this with more refined clinical observations. Our hope is that by the time of our next review, the use of early, targeted immunotherapies is commonplace in these conditions and has had an appreciable impact on the long-term outcomes of patients. To integrate the emerging biological insights with our progress around patient-relevant outcomes and the limitations of currently available therapies, the field now needs to embrace experimental medicine approaches. Some classical clinical trials are underway but may be hampered by difficulty recruiting immunotherapy-naïve patients. Nevertheless, a large pipeline of exciting new treatments will likely enter clinical trials in the near future. If incorporated with further dissection of the underlying biology, these trials are likely to provide a foundation for patient treatments over the next decade.

## Data Availability

No new data were generated in this manuscript.
